# Is the SES and academic achievement relationship mediated by cognitive ability? Evidence from PISA 2018 using data from 77 countries

**DOI:** 10.3389/fpsyg.2023.1045568

**Published:** 2023-02-17

**Authors:** Björn Boman

**Affiliations:** Department of Education, Stockholm University, Stockholm, Sweden

**Keywords:** PISA, SES, cognitive ability, IQ, cognitive school skills

## Abstract

**Introduction:**

Earlier research has suggested that that the international large-scale assessment, PISA (Programme for International Student Assessment), may be looked upon as a form of school test that is mostly explained by participating students’ socioeconomic status, non-cognitive factors, and various school factors, whereas another strand of research focuses on the similarities between PISA and cognitive ability assessments such as IQ tests. The latter position does also highlight the strong relationships between PISA scores and IQ test scores, typically aggregated to the country level. The current article adds to this scholarly debate by examining the latest PISA survey from 2018.

**Methods:**

Correlation, regression, moderator, and mediation analyses were run for aggregated country-level data (N = 77) from PISA 2018.

**Results:**

The results indicate that PISA scores indeed are strongly associated with both cognitive ability test scores and socioeconomic status indicators such as average annual national income.

**Discussion:**

A more nuanced position suggests that PISA should be labelled a test that measures cognitive school skills rather than a proxy of national IQ, as the link between country-level abilities and school age abilities is neither causal, nor theoretically and empirically palpable, yet partly robust.

## Introduction

A long-lasting debate within educational research concerns which factors are the strongest predictors of educational achievement [e.g., grades, test scores, and international large-scale assessments (ILSAs)]. Recent research (Guez et al., 2018; Vazsonyi et al., 2022; Boman, 2022c) suggests that cognitive ability is more important than conscientiousness or self-control, followed by socioeconomic status (SES). Others have stressed the near-equal magnitude of cognitive ability and conscientiousness for school achievement (Poropat, 2009; O’Conell and Marks, 2022). Yet others have focused more on interrelated non-cognitive abilities such as conscientiousness, grit (passion and perseverance), growth mindset (the belief that intelligence is malleable), and self-efficacy (individual’s belief in their own capacities; e.g., Duckworth et al., 2007; Cheung, 2017; Thorsen et al., 2021; Affuso et al., 2022), or the relations between non-cognitive abilities and cognitive abilities with regard to IQ tests and scholastic achievement tests (Borghans et al., 2008, 2016). This has led some researchers (e.g., O’Conell and Marks, 2022) to suggest a different theoretical framework that emphasizes the genetic transmission of cognitive abilities from parents to children rather than family SES such as economic and cultural resources exerting a strong direct influence on academic achievement, a view that is common with regard to the Program for International Student Assessment (PISA). Thus, it seems that the Organization for Economic Cooperation and Development (OECD) exaggerates the SES effects within and between classrooms, schools, and countries, and it is likely that cognitive ability and conscientiousness are more important factors (Deary et al., 2007; O’Conell and Marks, 2022; Boman, 2022a). However, there is a dearth of studies that have focused on the mediating or moderating effects of cognitive ability on PISA performance relative to SES and cognitive ability. Moreover, several studies only included a limited number of samples from earlier PISA waves when participants were fewer. The current study analyzed these relationships at the cross-national level. Specifically, 77 PISA participants from the PISA 2018 wave were analyzed with respect to the relations between PISA scores across mathematics, reading, and science and cognitive ability and SES. The following research questions were addressed:

1. Does SES mediate or moderate the relationships between cognitive ability and PISA achievement?
2. Does cognitive ability mediate the relationships between aggregated SES and PISA achievement?

## Theoretical background

### PISA as an international large-scale assessment for 15-year-old

Program for International Student Assessment, which was first administered in 2000, is a triennial, on average 2-h long, low-stake standardized assessment designated to test students within countries, as well as to make international comparisons between school systems (i.e., groups of students) and examine changes within education systems. Individuals complete test questions in three domains: reading, math, and science. In each cycle, one of these three subjects is the main subject, which is given somewhat greater analytical attention compared to the others. In addition, later PISA studies (2012 onward) have included problem solving and financial literacy add-ons for some countries. In PISA 2018, global competence was a novel domain. The test questions cover a rather broad spectrum of domain-specific cognitive skills (e.g., related to mathematics) and several levels of difficulty (i.e., the questions are situated at various levels of difficulty). The PISA surveys are conducted on two-stage stratified samples of students enrolled in lower secondary or, to a lesser extent, upper secondary schools. The included students are aged between 15 years and 3 months and 16 years and 2 months. The two-stage sampling strategy implies that schools are sampled first, and then students are sampled within schools (OECD, 2019a,b,c).

Furthermore, the aim of the PISA test is to pinpoint a broad coverage at the group level of the distribution of ability in different subject domains. Student responses to individual test items are used to impute the likelihood that such a student, and similar students, will able to solve items at the same, higher, or lower level of difficulty and with similar content. Plausible values make it possible to account for the probabilistic constitution of assessments of various levels of “unobservable” latent student abilities based on the observed response patterns (OECD, 2009, 2019c). The plausible values, as well as the different sub-tests (e.g., mathematics and reading) within PISA, are highly correlated (e.g., Saalik et al., 2015; Boman, 2022a). PISA scales are divided, in each domain assessed, into six or more proficiency levels. Each proficiency level is characterized in relation to the knowledge and skills that students demonstrate in the test.

In PISA 2018, 79 countries or urban regions, consisting of approximately 612, 000 students, participated. The OECD average scores are typically set at 500 with 100 as a standard deviation (e.g., Jerrim et al., 2018), but in PISA 2018, the average for all three tests was 488.5 (OECD, 2019b). As mentioned earlier, the PISA sample sizes do typically consist of approximately 5,000 individuals but countries such as Australia (14,273), B-J-S-Z (12,058), Brazil (10,691), the United Kingdom (13,808), and United Arab Emirates (19,277) had considerably larger sizes. Iceland had only 3,294 and Macao 3,775 students, which reflects their small populations (OECD, 2019c, pp. 362–363). In this survey, Japan, South Korea, and Estonia had the highest results among the OECD countries (OECD, 2019a).

### Cognitive ability, SES, and PISA

According to Gottfredson (1997), cognitive ability constitutes a general ability rather than narrow academic skills that includes problem solving and appropriate application of knowledge. Cognitive ability is typically measured by brief or extensive IQ tests. In comparison, PISA measures a set of cognitive skills in three domains, mathematics, reading, and science, and their relations to real life situations and contexts in the 21st century (OECD, 2019a). Even though the PISA tests are not particularly related to curricular knowledge, they are still more narrow and school-oriented than cognitive ability tests (Rindermann and Baumeister, 2015; OECD, 2019a).

As Avvisati (2020) and Lee and Borgonovi (2022) have emphasized, socioeconomic status is a multifaceted concept that rests on several, sometimes theoretically contradictory, assumptions. Some notions of SES focus more on the possession of cultural artifacts among middle-and upper-class families. These in turn, are expected to lead to social reproduction, as intellectually oriented culture, which is shown by students in school contexts, is awarded by teachers in terms of higher school results (e.g., Bourdieu and Passeron, 1977; Bourdieu, 1984). Later studies (e.g., refer to Milne and Aurini, 2015, for an overview) have focused more on the interplay between high-SES students and the abilities that the students obtain in schools. Moreover, a similar strand of research has focused on the characteristics of the family as regards cognitive skills and parental education (e.g., Willms, 2002; Myrberg and Rosén, 2009).

Other perspectives accentuate the cognitive growth of students when involved, often more highly educated parents read to their children and/or spend more resources on their children compared to families with lower SES (Turkheimer et al., 2003; Engelhardt et al., 2018; Falk et al., 2021). High-income families may send their children to better schools and spend resources on out-of-school learning such as private tutoring. Even though the SES effects on academic achievement differ between countries and cultures, it is a generic pattern that higher SES is associated with higher academic achievement, whether directly or indirectly (e.g., Sirin, 2005; Bray, 2006; Sackett et al., 2009; Kim and Lee, 2010; Kim, 2019; Lee and Borgonovi, 2022).

Marks and O’Connell (2021) underline the potential confounding effect of the SES–academic achievement theory. Specifically, when controlling for parents’ and children’s cognitive abilities, as well as prior achievement, much of the SES effect becomes negligible. However, as the authors underline, the cognitive ability cannot completely explain SES, and nor can SES explain the entirety of cognitive ability. Hence, these two constructs may be estimated separately but should be included in the same multivariate models (e.g., Boman, 2022c).

International large-scale assessments (ILSAs) have been studied since the 1960s (Boman, 2022b). Many researchers have assumed that these measure a latent ability among students (e.g., Hanushek, 1979) but tests such as PISA have not been labeled with a single term. The concept of “literacy” is used regarding the assessment and interpretation of students’ performance in the three domains. “Literacy” specifically denotes “students’ capacity to apply knowledge and skills in key subjects, and to analyze, reason and communicate effectively as they identify, interpret and solve problems in a variety of situations” (OECD, 2017a, p. 13). The PISA achievement term used by the OECD is literacy (Rindermann and Baumeister, 2015) whereas, for example, Hanushek and Woessman (2008) used the term cognitive skills, and Lynn and Mikk (2007), Lynn and Vanhanen (2012), and Lynn and Becker (2019) defined the sum of achievement as national IQs (NIQs). The strong correlations between cognitive ability tests (e.g., IQ tests) and ILSAs indicate that these tests, to different extents, measure the same general cognitive abilities such as verbal and mathematical reasoning (Rindermann, 2007, 2018; Rindermann and Ceci, 2009; Rindermann and Baumeister, 2015). Rindermann and Baumeister (2015) stress that PISA tests in all domains require good reading skills. Later research such as Flores-Mendoza et al. (2021) and Pokropek et al. (2022) confirmed the strong relationships between PISA and general cognitive ability. Hence, when aiming to understand what drives PISA scores, it is pertinent to examine cognitive abilities aggregated to the country level (Boman, 2022a).

What remains unclear, however, is how these relationships should be interpreted in a meaningful way. For example, it is not only the case that cognitive abilities are affected by national development (Hunt and Wittman, 2008) but that PISA scores specifically are more influenced by reading abilities than many IQ tests (Rindermann and Baumeister, 2015). Moreover, at the school level, there are effects at play that seem to transcend individual level differences (e.g., Liu et al., 2022). Hence, it is perhaps more appropriate to label these cognitive skills as cognitive school skills rather than national IQ tests. This means that international cognitive tests, such as PISA are situated in a school context among school students within a particular age range, whereas IQ tests, such as Wechsler Adult Intelligence Scale (WAIS) and Wechsler Intelligence Scale for Children (WISC) are distributed across different age groups among children (6–15-year-old) and adults (16–65-year-old; c.f., OECD, 2009; Weiss et al., 2010; Kaufman et al., 2016). The PISA age is greatly affected by school knowledge (Rindermann, 2018) and contextual school factors (Yang Hansen et al., 2022). Therefore, it is different from IQ tests (especially among adults) that cover individual abilities which are greatly influenced by genetics (Engelhardt et al., 2018). Thus, PISA is conceptually an international cognitive skills test but not an international IQ test.

The rather strong cross-sectional relation between SES and PISA scores, however, inflated by the cross-sectional study design and omitted variable bias (e.g., Marks and O’Connell, 2021; Boman, 2022a), requires a researcher to include SES as an important predictor at the country level. Because SES is a crucial variable at the individual level, it is likely that it also has an effect at the country level with regard to PISA scores (Burhan et al., 2017; Flores-Mendoza et al., 2021). This relationship may be captured by logged national average income or a proxy, such as GDP *per capita*.

As slightly touched upon above, some researchers propose that high cognitive abilities among populations lead to high GDP *per capita* (at the national level) or average salaries (at the individual level; e.g., Lynn and Mikk, 2007), whereas others suggest that high abilities reflect a high degree of development at the country level, as indicated by GDP *per capita* (e.g., Hunt and Wittman, 2008; Wicherts et al., 2010; Burhan et al., 2017; Komatsu and Rappleye, 2017, 2019; Rindermann, 2018; Daniele, 2021).

While both theoretical assumptions might be true, to some extent, it might be suggested that cognitive abilities constitute a predominant comparative advantage (Boman, 2020), as they reflect an underlying construct that is more similar to school achievement, PISA in particular (Marks and O’Connell, 2021). This implies that there is likely a stronger relationship between PISA scores and cognitive ability scores than between PISA scores and average income. Hence, by examining cognitive ability levels among countries, it is possible to discern a strong link to the scores in international scholastic assessments. The same goes for SES but this relationship seems to be somewhat weaker (e.g., Rindermann, 2018; Flores-Mendoza et al., 2021).

Similar to how cognitive ability may decrease (i.e., negative selection for high-SES children) and increase simultaneously (i.e., the Flynn effect, the trend of increasing IQ test scores, refer to, e.g., Flynn, 2012), it is possible that cognitive development and economic development are interrelated patterns (Levin, 2009; Boman, 2021a,b). More specifically, countries require some degree of average general cognitive ability to develop. However, economic development, in the next step, improves the lives of families, schools, and the country in its entirety, which then improves, to some extent, the cognitive abilities and school achievement (Rindermann, 2018; Komatsu and Rappleye, 2019). Based on earlier research and theory, the following hypotheses are suggested:

*H1*: It is assumed that the correlations between NIQ and PISA scores will be large, that is, above *r* = 0.70, and medium to high for average income and PISA, above *r* = 0.40 (Dancey and Reidy, 2007).

*H2*: Furthermore, it is likely that cognitive ability partially mediates or moderates the relationship between SES and PISA scores (Marks & O'Connell, 2021).

*H3*: Because of the comparatively smaller correlation between SES and cognitive ability (Marks & O'Connell, 2021; Boman, 2022c), it is hypothesized that SES does not mediate the relationship between cognitive ability and PISA scores.

## Methods

### Data, variables, and procedures

Data for 77 of 79 PISA participants in the 2018 survey were retrieved from OECD (2019a). A few countries did not have complete data on all sub-tests and were, therefore, excluded. This was considered quite unproblematic from a statistical viewpoint, and no missing data imputations were conducted regarding the analyses.

An aggregated sum score across mathematics, reading, and science, at the country level, was created. This is appropriate as this provides a more comprehensive score of the country’s overall PISA performance (Rindermann, 2018). The independent variable average income for 2018 was log-transformed and such data were retrieved from World Bank (2022). However, for Beijing, Jiangsu, Shanghai, and Zhejiang in China and Moscow in Russia, city level data were retrieved from Statista (2022). That is because major urban regions in China (e.g., Boman, 2022b) and Russia (e.g., Griogoriev et al., 2016) have higher test scores than rural regions and smaller cities, and it is, therefore, inappropriate to use country level data in those two contexts.

Data on students’ cognitive abilities, aggregated to the country level [i.e., national (IQ NIQ)], were retrieved from Lynn and Becker (2019). This self-published book consists of a collection of mostly peer-reviewed cognitive ability scores that cover a substantial share of the world’s countries. Lynn and Becker (2019) used very specific procedures to calculate their national IQ scores, including all available IQ samples and corrections for sample size and the Flynn effect (i.e., the tendency that test scores have risen throughout the 20th century). This resulted in scores that were typically lower than the raw scores from earlier cognitive ability studies because they had to remove IQ points due to sample size and sampling year. One may notice that with older standardizations, countries such as Romania (Iliescu et al., 2016) and South Korea (Kwak, 2003) have higher scores than which is the case in Lynn and Becker (2019). Hence, the scores should be looked upon as contextualized and “corrected” scores rather than raw scores.

However, the “SAT weightings” that were included in the composed IQ values in Lynn and Becker (2019) were, in the current study, removed to avoid a confounding effect, meaning that older scores from, for example, PISA and TIMSS correlate with recent PISA scores. Here, the focus was on the strict NIQ–PISA relationships. Whenever a few jurisdictions had no cognitive ability data (*N* = 4, Kosovo, Macau, Moldova, North Macedonia), the author estimated the scores by averaging the scores from two neighboring countries (Lynn and Vanhanen, 2012). Data for PISA scores, average income (USD), and NIQ scores are shown in Table 1.

**Table 1:** Descriptive statistics PISA 2018.

Country	PISA scores	Income (USD)	NIQ
China (B-S-J-Z)	578,8	18,720	104,97
Singapore	556,3	48,345	104,58
Macao	542,3	27,192	104,97
Hong Kong	530,7	56,000	106,06
Estonia	525,3	19,058	98,58
Japan	520	31,070	107,41
South Korea	519,7	27,237	97,37
Canada	516,7	37,637	97,9
Taiwan	516,7	14,273	108,69
Finland	516,3	40,903	99,31
Poland	513	13,030	94,92
Ireland	504,7	38,274	89,94
Slovenia	503,7	20,763	98,6
UK	503,7	36,247	98,23
New Zealand	502,7	35,281	99,01
Netherlands	502,3	44,714	100,19
Sweden	502,3	46,250	94,96
Denmark	501	52,851	96,68
Germany	503	40,745	102,33
Belgium	500	39,065	97,07
Australia	499	43,950	99,52
Switzerland	498	63,798	97,26
Norway	496,7	65,890	99,51
Czechia	495,3	17,423	90,62
United States	495	53,666	95,86
France	493,7	34,902	97,02
Portugal	492	18,806	89,71
Austria	491	41,593	97
Latvia	487,3	13,751	91,14
Russia	481,7	15,420	92,95
Iceland	481,3	58,346	100,5
Lithuania	479,7	13,291	94,53
Hungary	479,3	13,114	99,21
Italy	477	28,814	91,66
Luxembourg	476,7	69,216	97,05
Belarus	472,3	5,205	101,6
Croatia	471,7	12,674	93,92
Slovakia	469,3	15,795	95,32
Israel	465	36,396	90,57
Turkey	462,7	7,991	86,66
Ukraine	462,7	2,728	88,61
Malta	459	16,938	90,72
Greece	453,3	16,529	86,45
Serbia	442,3	5,869	87,82
Cyprus	438	24,780	95,51
Chile	437,7	12,152	89,85
UAE	433,7	40,079	79,48
Malaysia	431	7,796	86,05
Romania	428	9,947	83,11
Bulgaria	426,7	7,755	87,1
Moldova	424,4	3,881	84,61
Uruguay	423,7	14,745	86,22
Brunei	423	31,628	82,25
Montenegro	422	8,079	89,37
Albania	419,7	4,319	89,37
Jordan	416	4,045	77,97
Mexico	416	7,527	90,44
Costa Rica	414,7	11,139	88,34
Qatar	413,3	49,682	85,58
Thailand	412,7	5,587	89,78
Colombia	405,3	5,519	85,95
Kazakhstan	402,3	6,571	84,27
Azerbaijan	402,3	5,547	84,27
Bosnia	402,3	5,054	90,92
Peru	401,7	5,711	85,39
Brazil	400,3	7,762	85,23
North Macedonia	400	4,966	89,37
Argentina	395	9,812	93,85
Georgia	387	3,864	84,27
Saudi Arabia	386	18,892	78,48
Indonesia	382	3,080	89,28
Lebanon	376,7	6,550	83,3
Morocco	368	2,825	68,73
Panama	365	13,189	88,34
Kosovo	361,3	500	89,37
Philippines	350	3,284	92,47
Dominican Republic	334,3	7,216	89,15

The author decided not to include more covariates in the main analyses, as many factors at the national level are highly intercorrelated, such as GDP *per capita*, teacher salaries, and democracy index, or simply are not good predictors of school achievement such as PISA scores at the country level (Boman, 2022a). Moreover, many aggregated school level variables are missing outside the OECD (OECD, 2021). Hence, it would be inappropriate to include data for only approximately 31 out of 77 countries and jurisdictions.

### Analytical strategy

The first step was to examine the bivariate relationships between the three variables, followed by an ordinary least squares (OLS) regression analysis. Observing bivariate correlations is an appropriate first step prior to conducting regression analysis (Field, 2018). Pearson’s correlation of *r* = 70 is the threshold for a high correlation within the psychology field, according to Dancey and Reidy (2007).

At a later step, moderation and mediation analyses were conducted using SPSS 26 for the moderator analysis and the SPSS extension PROCESS (Hayes, 2020) for the mediation analysis and an additional moderator analysis. Specifically, a moderation model, which included a cognitive ability*SES variable, was included. Due to potential problems with multicollinearity, the two variables of the interaction term were mean-centered and aggregated (Field, 2018). The step is required to answer both the research questions as these are concerned with the moderation and mediation between cognitive ability, SES, and PISA scores.

According to Zhao et al. (2010), the basic assumption with regard to mediation models is that the direct effects from the *x* variable (i.e., the independent variable) must be statistically significant and that is also the case with the mediator (m). Then the direct and indirect effects of the predictors estimate the model fit (Cheung, 2009; Zhao et al., 2010).

Due to the fact that this is a limited sample at the country level, no additional significance tests were run (e.g., Wasserstein et al., 2019). However, due to the potential magnitude of the effect sizes (e.g., the standardized beta coefficients), it was expected that both SES and cognitive ability would be statistically significant at the 5% level in the regression models (Cohen, 1988; Field, 2018; Komatsu and Rappleye, 2019). The author used *p* = 0.05 as the significance level threshold (e.g., Cohen, 1988; Field, 2018).

In addition, a robustness model was conducted. Specifically, an additional control variable, democracy index scores (The Economist Intelligence Unit, 2018), was added to the regression analysis.

## Results

The bivariate correlations (refer to Supplementary information) resulted in large correlations, *r* = 0.766 (value of *p* < 0.001) for NIQ and PISA and *r* = 0.699 (value of *p* < 0.001) for average national income and PISA. In statistical terms, that means that approximately 59% of the PISA scores can be explained by the average aggregated national IQ scores and that approximately 49% of PISA may be explained by average national income. On the other hand, these are merely preliminary results.

However, the regression analysis (refer to Table 2) showed a more realistic *R^2^* value, with a model that explained approximately 70% of the variance. The standardized beta coefficient of cognitive ability (β = 0.548) was, as expected, larger than the beta coefficient for income (β = 0.401).

**Table 2:** Results of a regression analysis.

Variable	B	β	*SE*
Constant^*^	−115.776		43.753
Income^*^	21.792	0.401	4.048
Cognitive ability^*^	3.926	0.548	0.538

*R*^2^: 0.703. Table 2 shows the relationship between PISA achievement (dependent variable) and average annual national income and aggregated cognitive ability scores (NIQ) for *N* = 77 countries and jurisdictions that participated in PISA 2018. ^*^Value of *p* < 0.001.

The moderator analysis which was conducted in SPSS shows that the interaction term was not statistically significant. The moderator analysis in PROCESS, with 5,000 bootstrapped cases, confirms these results (refer to Supplementary information).

The mediation analysis (refer to Table 3) indicates that neither SES nor cognitive ability mediates the PISA results, even though the total direct effects were significant. In conjunction with the results from the moderator analyses, the findings indicate that PISA scores are influenced by the linear effects of both the cognitive ability level and economic development level of a given country. Because these two variables are moderately intercorrelated, *r* = 0.537, it is also likely that they influence each other. The magnitude of such interrelationships is not possible to assess here but has been discussed in much previous research. The relationships are most likely quite complex and bi-directional (e.g., Hunt and Wittman, 2008; Burhan et al., 2017; Lim et al., 2018; Rindermann, 2018; Komatsu and Rappleye, 2019; Daniele, 2021).

**Table 3:** Results of mediation analysis.

Mediator	Coefficients	SE	LLCI	ULCI
Cognitive ability				
Ind. effect	0.0009	0.0002	0.0006	0.0013

*R*^2^: 0.646. LLCI, lower level confidence interval; ULCI, upper level confidence interval. *N* = 77. The mediation model shows how aggregated cognitive ability (NIQ, national IQ) may mediate the results between PISA scores and average national income.

As a robustness check, democracy index data from the Economist Intelligence Unit’s annual report, specifically 2017 (The Economist Intelligence Unit, 2018), was included as a continuous predictor alongside national income and NIQ. Even though democracy is moderately correlated with PISA, NIQ, and income, this contextual factor at the cross-national level did not add to the model, nor did it affect the overall relationships (refer to Supplementary information). Hence, the results of the regression analyses seem robust.

## Discussion

As the bivariate correlations, regression models, and earlier research show (e.g., Rindermann, 2007; Flores-Mendoza et al., 2021; Boman, 2022a), there are strong links between PISA and cognitive ability, and between PISA and SES, thus overall confirming the first hypothesis. Specifically, the SES–PISA relationships, in this case, are situated at the threshold for a strong rather than moderate effect size (*r* = 0.699, where 0.70 is the threshold for a high correlation, refer to Dancey and Reidy, 2007). However, these correlations may not be strong enough to equate cognitive ability, as in IQ, with PISA scores. Hence, PISA is not a national IQ test. Furthermore, due to age differences among country IQ samples summarized in Lynn and Becker (2019), it appears inappropriate to draw too far-reaching links between IQ test results and current PISA achievement. Hence, as said, one may prefer a different term, such as cognitive school skills. These cognitive school skills do likely reflect the overall ability level within a country, at least on average, but there are other relevant factors that influence PISA achievement, such as economic development, representativeness of the PISA samples (Boman, 2022b), and non-cognitive factors that are typically difficult to measure at the country level. Test motivation in low stakes tests may have some effect, but it is often negligible (e.g., Rindermann and Ceci, 2009; Balart et al., 2018). At the school level, there are also other factors at play such as instructional quality and its interaction with SES (e.g., Liu et al., 2022).

With regard to the other hypotheses, no moderating or mediating effects were found for cognitive ability’s potential influence on SES and vice versa in relation to PISA achievement. Hence, the second hypothesis was not confirmed by the analyses. The third hypothesis, which suggested that SES does not mediate the relationship between cognitive ability and PISA scores, is partly confirmed, but the correlation between SES and cognitive ability is moderate and thus larger compared to what much individual level data show (e.g., Marks and O’Connell, 2021; Boman, 2022c).

This study has several limitations. As mentioned earlier, due to the cross-sectional design, it is not possible to control for participating students’ previous academic achievement, effect sizes will be skewed upward, and causal relationships not possible to discern. Moreover, due to the aggregated data, it is not possible to make inferences at the individual or school level (Boman, 2022d). Some may still consider the Lynn and Becker (2019) data set to be unreliable and misguiding (Sear, 2022). The author has accounted for some of these problems and interpreted the correlations on the basis of the partial mismatch between age and sampling year. Regarding more complex validity and reliability issues such as measurement invariance among individual level country samples in either PISA assessments or cognitive ability tests (e.g., Wicherts and Dolan, 2010; Odell et al., 2021; Ding et al., 2022), one should be careful to naively accept the aggregation of IQ scores. However, PISA scores, on the other hand, are representative, and the descriptive statistics show that some countries consistently outperform others (e.g., OECD, 2019a). Thus, the PISA results confirm the IQ aggregates in that regard.

Furthermore, the data are limited to only a single wave of PISA scores, whereas other studies have included more waves (e.g., Rindermann and Ceci, 2009; Becker et al., 2022). The results cannot be generalized to all countries, and while improved in terms of participation, the PISA 2018 survey only covers approximately 40% of the world’s countries and jurisdictions. The fact that only average national income is used to indicate SES might also be problematic as parental education might be a more appropriate indicator in that respect (e.g., Sackett et al., 2009; Avvisati, 2020; Marks and O’Connell, 2021). Also, migration background and taking the test in another language than one’s mother tongue matter (Meunier, 2011). However, the average income is crucial as a country level indicator simply because it captures both overall SES and overall economic development.

Future research may include more waves of PISA and perhaps consist of school level covariates in multi-level model analyses, which are often used for secondary analysis of PISA (e.g., Huang et al., 2019). It might also be important to include TIMSS (Trends in Mathematics and Science Study), which is more related to curricular features in each participating country (Rindermann and Baumeister, 2015), in order to distinguish if these country-level patterns are confounded by overall national levels of ability and economic development. With regard to future PISA tests, it would be very useful if the participants, or at least fractions of the participants in all or most countries, conduct brief cognitive ability tests (i.e., IQ tests). Only then could the relative impact of IQ be comprehensively compared to SES and non-cognitive effects as regards both individual level and country level differences (Boman, 2022a). Furthermore, if the OECD manages to include data from all PISA participants, more country level covariates may be included.

## Data availability statement

The original contributions presented in the study are included in the article/Supplementary material, further inquiries can be directed to the corresponding author.

## Author contributions

The author confirms being the sole contributor of this work and has approved it for publication.

## Conflict of interest

The author declares that the research was conducted in the absence of any commercial or financial relationships that could be construed as a potential conflict of interest.

## Publisher’s note

All claims expressed in this article are solely those of the authors and do not necessarily represent those of their affiliated organizations, or those of the publisher, the editors and the reviewers. Any product that may be evaluated in this article, or claim that may be made by its manufacturer, is not guaranteed or endorsed by the publisher.
