# Supplementary material for: Is the SES and academic achievement relationship mediated by cognitive ability? Evidence from PISA 2018 using data from 77 countries
Source: Front Psychol. 2023 Feb 17;14:1045568. doi: 10.3389/fpsyg.2023.1045568 (PMC9994354; doi:10.3389/fpsyg.2023.1045568)
Supplement: Supplementary file 1 [file Table_1.DOCX]

**Is SES-academic achievement mediated by cognitive ability: Appendix A**

| **Correlations between PISA scores, NIQ, and national income** | | | | |
| --- | --- | --- | --- | --- |
|  | | PISA scores | NIQ | Income |
| PISA scores | Pearson Correlation | 1 | ,766^**^ | ,699^**^ |
|  | Sig. (2-tailed) |  | ,000 | ,000 |
|  | N | 77 | 77 | 77 |
| NIQ | Pearson Correlation | ,766^**^ | 1 | ,537^**^ |
|  | Sig. (2-tailed) | ,000 |  | ,000 |
|  | N | 77 | 77 | 77 |
| lncome | Pearson Correlation | ,699^**^ | ,537^**^ | 1 |
|  | Sig. (2-tailed) | ,000 | ,000 |  |
|  | N | 77 | 77 | 77 |
| **. Correlation is significant at the 0.01 level (2-tailed). | | | | |

**Moderation analysis**

|  | | | | | | | | |
| --- | --- | --- | --- | --- | --- | --- | --- | --- |
| Model | | Unstandardized Coefficients | | Standardized Coefficients | t | Sig. | Collinearity Statistics | |
|  |  | B | Std. Error | Beta |  |  | Tolerance | VIF |
| 1 | (Constant) | -12,703 | 78,127 |  | -,163 | ,871 |  |  |
|  | Income | ,003 | ,003 | 1,036 | ,969 | ,336 | ,004 | 237,451 |
|  | NIQ | 4,886 | ,868 | ,682 | 5,630 | ,000 | ,328 | 3,053 |
|  | Income*IQ | -2,321E-5 | ,000 | -,786 | -,704 | ,484 | ,004 | 259,333 |
| a. Dependent Variable: PISA. *R^2^*: .649 | | | | | | | | |
